# Real-Time Updating High-Order Extended Kalman Filtering Method Based on Fixed-Step Life Prediction for Vehicle Lithium-Ion Batteries

**DOI:** 10.3390/s22072574

**Published:** 2022-03-28

**Authors:** Jincheng Wang, Chenglin Wen

**Affiliations:** 1 School of Automation, Hangzhou Dianzi University, Hangzhou 310018, China; jcw@hdu.edu.cn; 2 School of Automation, Guangdong University of Petrochemical Technology, Maoming 525000, China

**Keywords:** lithium-ion battery, remaining useful life, iterative recursion, multi-dimensional taylor network, high-order extended Kalman filter, multi-step prediction

## Abstract

Lithium-ion batteries have become an important power source in low-carbon transportation energy, and the safe operation and remaining useful life prediction are of great significance. Aiming at the shortcomings of existing methods, such as low prediction accuracy and a short prediction period, this paper proposes a real-time update high-order extended Kalman filter method based on fixed-step life prediction for vehicle lithium batteries based on the principle of combining models and data. First, the state model describing the parameters in the dynamic energy attenuation model is established, and the energy attenuation model is regarded as the observation model of the system to meet the requirements of establishing the Kalman filter. Secondly, the multi-step prediction equation of the state model is established by iterative recursion. At the same time, the multi-step prediction equation between the existing energy output value and the future output value is established based on the multi-dimensional Taylor network (MTN). The multiplicative noise term introduced in the dynamic modeling process is regarded as the hidden variable of the system to meet the requirements of establishing the multi-step linear predictive Kalman filter. Finally, the effectiveness of the new method is verified by digital simulation examples.

## 1. Introduction

In order to meet the demand of global carbon neutrality and realize the development of low-carbon transportation, the creation of electric vehicles, electrified trains and electric aircraft has contributed to low-carbon transportation. The production and scrap process of lithium-ion batteries with high storage energy density, low self-discharge rates and strong adaptability to high and low temperatures will not produce harmful heavy metals and substances, which is in line with the concept of low-carbon environmental protection. However, the production conditions and costs are relatively high, and thus its health status and remaining useful life are related to the operation status and safety of the transportation equipment. Therefore, the remaining useful life prediction of lithium-ion batteries is particularly important [1].

The remaining useful life (RUL) of a lithium-ion battery is defined as the number of charging and discharging cycles remaining before the performance deteriorates to the rated fault threshold [2]. In the existing literature, the prediction and estimation methods of lithium-ion batteries can generally be divided into model-based methods, data-driven methods and hybrid methods [3]. The model-based method is highly dependent on the degradation model and failure analysis of lithium-ion batteries. The existing filtering methods, such as extended Kalman filter (EKF) [4], unscented Kalman filter (UKF) [5] and particle filter (PF) [6], can effectively predict and estimate the health status and RUL of lithium-ion batteries in the future.

These filtering methods achieve better prediction accuracy by updating the internal parameters of the battery online. For example, a novel technique, which integrates a recursive total least squares (RTLS) with an state-of-charge (SOC) observer, was proposed to enhance the online model identification, and SOC estimation [7], a novel constant-voltage (CV) phase reconstruction method combining Q-V modeling and open-circuit voltage (OCV) estimation iteratively was proposed to predict the CV capacity authentically based on the available partial CV data. The extracted CV capacity is further used to estimate the battery state-of-health (SOH) precisely [8].

Aiming at the problem that the model-based method is difficult to apply, Zheng et al. proposed a differential voltage analysis method based on a general battery model and used EKF and PF to realize the prediction and estimation of RUL [9]. Zhang et al. proposed a particle filter method based on exponential model to predict the RUL of lithium-ion batteries. Compared with the autoregressive integrated moving average model, this method had better prediction performance [10]. After that, Lin et al. changed the double exponential empirical degradation model reduced the number of parameters of this model, and proposed a PF method based on an autoregressive time series model [11].

In addition to improving the lithium-ion battery degradation model, there are also methods to improve the filters. The extended Kalman particle filter improves the problem of particle degradation in the PF algorithm and improves the prediction accuracy. The improved unscented particle filter based on the Markov chain Monte Carlo method can also suppress the particle degradation problem in the standard PF algorithm [12,13].

However, due to the lack of physical explanation and understanding of the degradation process of lithium-ion batteries, complex and accurate mathematical models may not be established, and accurate prediction results are naturally not available. On the contrary, when the degradation mechanism fails or is unknown, as long as there is sufficient historical data for the lithium-ion battery, the RUL can be predicted using a data-driven method [14,15].

Different data-driven RUL prediction methods have been introduced, including autoregressive models (ARmodels), artificial neural networks (ANNs), support vector machines (SVMs) and relevance vector machines (RVMs) [16]. These have different advantages and disadvantages. The prediction method based on the AR model is simple; however, its prediction accuracy is low. Lin et al. explained the degradation structure by the variation of regression line length and slope, and proposed a reliable prediction method using the combination model of the autoregressive model and the survival regression model [17].

The prediction method based on an artificial neural network has sufficient prediction accuracy; however, there are problems, including a lack of robustness, a large amount of calculations and over-fitting. Using false nearest neighbors and a hybrid neural network effectively solves these problems [18]. The prediction method based on support vector machine can achieve high prediction accuracy only using small sample training; however, its parameter identification is challenging. Yang et al. proposed an algorithm based on statistical knowledge, which has the advantages of global optimization, high precision and a strong generalization ability [19].

Based on the multi-level computing support vector machine method, Patil et al. made the RUL prediction of lithium-ion batteries faster and more accurate, and their method can be used for real-time vehicle RUL estimation of electric vehicle battery packs [20]. The method based on relevance vector machine does not require a large number of samples; however, its long-term prediction ability is weak with poor stability.

Cheng et al. proposed an optimization algorithm based on a combination of the broad learning system (BLS) and relevance vector machine (RVM), which inputs different features into the BLS network, sets different prediction starting points, and finally outputs the RUL prediction of the hybrid model. This algorithm improves the shortcomings of the original RVM algorithm and has stronger long-term prediction ability and generalization ability [21]. When introducing the above data-driven method, the deep-learning method is also excellent.

The deep-learning method has become the main data method to solve the RUL prediction of lithium-ion batteries in recent years. Recurrent Neural Networks (RNNs) are very effective for this sequential battery data, which can mine the temporal and semantic information in the data. For example, an artificial intelligence estimation method based on recurrent neural network and state space estimation technology are combined to estimate the RUL of lithium-ion batteries [22].

The long short-term memory (LSTM) neural network can deal with the time sequence information well, which is usually used to solve the long-term dependence problem, and this type of network has certain advantages for the RUL prediction of lithium-ion batteries [23]. However, the current technology is not satisfied with only one method—there are many hybrid deep-learning methods. A hybrid neural network was proposed in the literature that combines a convolutional neural network (CNN) and bidirectional long short-term memory network (Bi-LSTM) [24].

This approach has high reliability and prediction accuracy and can be applied to battery monitoring and prognostics. Similarly, in order to develop CNN and LSTM in depth for deeper information mining in limited data, Ren et al. proposed a new LIB RUL prediction method based on improved CNN and LSTM, namely Auto-CNN-LSTM [25]. At the same time, Zraibi et al. used the hybrid network of CNN, LSTM and Deep Neural Networks (DNN) to accurately predict the RUL of lithium-ion batteries, which proved that the hybrid network was superior to the single network [26].

In order to make full use of the mechanism characteristics of known models and the excellent computing power of data-driven algorithms, hybrid methods based on the combination of model and data-driven have developed rapidly in recent years, in particular, a hybrid method based on a filter-based data-driven method. Xiao et al. proposed a RUL prediction method for lithium-ion batteries based on UKF and a back propagation (BP) neural network. The BP neural network was used to compensate and correct the predicted value of the filter to obtain the estimated value [27].

In addition, UKF is used as the filtering method, and the future measurement value of lithium-ion battery is trained by RVM based on historical data to update the prediction error [28]. This does not waste the known mechanism model and historical data, and the prediction results are more robust and adaptable. Similarly, the literature also proposed a hybrid method based on UKF and relevance vector regression (RVR) also based on a similar theory [29].

The above-mentioned lithium-ion RUL prediction methods are based on one-step prediction. At present, there are few methods that can predict the RUL of lithium-ion batteries online in multiple steps. In one study [30], adaptive neuro-fuzzy inference systems, random forests, and the group method of data handling were used along with three strategies for multi-step prediction and prognosis. These methods use historical and current data to predict future measurements. If the RUL of lithium-ion batteries at multiple future moments can be predicted and analyzed online, it is of great significance for the monitoring and maintenance of traffic electronic equipment [31,32]. Using this idea and the recent RUL prediction method of lithium-ion batteries based on filtering and being data-driven, this paper establishes a multi-step prediction model and a training model based on a multi-dimensional Taylor network to predict future measurements. The main contributions are as follows:

- Based on the multi-step prediction model, the high-order extended Kalman filter is used to predict the estimation, and the predicted future measurements are integrated into the innovation update in the filter, thereby, realizing the multi-step advanced prediction of the RUL of the lithium-ion battery.
- This method achieves better prediction accuracy compared with the model-based method.
- It maintains the demand for smaller samples compared with the data-driven method.
- In addition, it also achieves multi-step prediction with strong robustness.

This paper is organized as follows: Section 2 introduces the background and basic problem setting. Section 3 introduces the derivation process of the multi-step prediction model. In Section 4, the process of multi-step prediction of future measurements based on a multi-dimensional Taylor network is analyzed. Section 5 establishes a hybrid method for predicting the RUL of lithium-ion batteries based on Section 2 and Section 3. In Section 6, the system simulation experiment proves the effectiveness of the proposed method. Section 7 is our summary.

## 2. Problem Description

In order to introduce the detailed process of the proposed multi-step prediction method, this paper considers the following conditions.

Regarding the degradation model of lithium-ion batteries, we assume that the degradation model of lithium-ion batteries is a first-order Markov process that can be expressed as follows:

(1) $$\left\{\begin{array}{c} x^{\left(1\right)}\left(k+1\right)=f\left(x^{\left(1\right)}\left(k\right),p\left(k\right)\right)+w^{\left(1\right)}\left(k\right)\\ y\left(k+1\right)=h(x^{\left(1\right)}\left(k+1\right))+v\left(k+1\right) \end{array}\right.$$

where f(·) and h(·) represent the state transition function and the measurement function, $x^{\left(1\right)}\left(k\right)\in R^{n}$ is the state variable, $y\left(k+1\right)\in R^{m}$ is the measurement variable. State noise $w^{\left(1\right)}\left(k\right)\in R^{n}$ and measurement noise $v\left(k+1\right)\in R^{m}$ are irrelevant zero-mean sequences, and the covariances are $Q^{\left(1\right)}\left(k\right)$ and R(k+1), respectively. The initial state $x_{0}^{\left(1\right)}$ is a random vector independent of the noise sequence, which satisfies

(2) $$E\left\{x^{\left(1\right)}\left(0\right)\right\}={\hat{x}}_{0}$$

(3) $$P_{0}^{\left(1\right)}=E\left\{\left[x^{\left(1\right)}\left(0\right)-{\hat{x}}_{0}\right]{\left[x^{\left(1\right)}\left(0\right)-{\hat{x}}_{0}\right]}^{T}\right\}$$

where $P_{0}^{\left(1\right)}$ is the initial error covariance matrix.

## 3. Multi-Step Prediction Modeling

Lithium-ion battery degradation model (1) is based on the previous time value of the state information and the current time measurement information to infer the next time state value. This is the current one-step prediction method based on the mechanism empirical model. In order to realize multi-step prediction, the mechanism empirical model of multi-step prediction should be established based on the mechanism empirical model of one-step prediction.

According to the one-step state model of Equation (1), the state model sequence from time k+1 to time k+q is obtained.

(4) $$\left\{\begin{array}{c} x^{\left(1\right)}\left(k+1\right)=A\left(k+1\right)x^{\left(1\right)}\left(k\right)+w\left(k\right)\\ x^{\left(1\right)}\left(k+2\right)=A\left(k+2\right)x^{\left(1\right)}\left(k+1\right)+w\left(k+1\right)\\ \vdots \\ x^{\left(1\right)}\left(k+q\right)=A\left(k+q\right)x^{\left(1\right)}\left(k+q-1\right)+w\left(k+q-1\right) \end{array}\right.$$

This can be expressed as

(5) $$\begin{array}{cc} \left[\begin{array}{c} x^{\left(1\right)}\left(k+1\right)\\ x^{\left(1\right)}\left(k+2\right)\\ \vdots \\ x^{\left(1\right)}\left(k+q\right) \end{array}\right] & =\left[\begin{array}{cccc} A(k+1) & A(k+2) & \cdots & A(k+q) \end{array}\right]\left[\begin{array}{c} x^{\left(1\right)}\left(k\right)\\ x^{\left(1\right)}\left(k+1\right)\\ \vdots \\ x^{\left(1\right)}\left(k+q-1\right) \end{array}\right]\\ \; & +\left[\begin{array}{c} w^{\left(1\right)}\left(k\right)\\ w^{\left(1\right)}\left(k+1\right)\\ \vdots \\ w^{\left(1\right)}\left(k+q-1\right) \end{array}\right] \end{array}$$

where A(·) is the state transition matrix.

The state at time *k* is sequentially combined into the state from k+1 to k+q, as shown in Equation (4).

(6) $$\begin{array}{c} x^{\left(1\right)}\left(k+1\right)=A\left(k\right)x^{\left(1\right)}\left(k\right)+w^{\left(1\right)}\left(k\right)\\ x^{\left(1\right)}\left(k+2\right)=A\left(k+1\right)A\left(k\right)x^{\left(1\right)}\left(k\right)\\ +A\left(k+1\right)w^{\left(1\right)}\left(k\right)+w^{\left(1\right)}\left(k+1\right)\\ \vdots \\ x^{\left(1\right)}\left(k+q\right)=\prod_{i=0}^{q-1}A(k+i)x^{\left(1\right)}\left(k\right)\\ +\sum_{l=0}^{q-2}\prod_{i=0}^{q-2-l}A\left(k+i+1\right)w^{\left(1\right)}\left(k+l\right)+w^{\left(1\right)}\left(k+q-1\right) \end{array}$$

Equation (4) is organized into a general matrix form, which is

(7) $$\begin{array}{cc} \left[\begin{array}{c} x^{\left(1\right)}\left(k+1\right)\\ x^{\left(1\right)}\left(k+2\right)\\ \vdots \\ x^{\left(1\right)}\left(k+q\right) \end{array}\right] & =\left[\begin{array}{cccc} A(k) & A(k+1)A(k) & \cdots & \prod_{i=0}^{q-1}A(k+i) \end{array}\right]x^{\left(1\right)}\left(k\right)\\ \; & +\left[\begin{array}{c} w^{\left(1\right)}\left(k\right)\\ A\left(k+1\right)w^{\left(1\right)}\left(k\right)+w^{\left(1\right)}\left(k+1\right)\\ \vdots \\ \sum_{l=0}^{q-2}\prod_{i=0}^{q-2-l}A\left(k+i+1\right)w^{\left(1\right)}\left(k+l\right)+w^{\left(1\right)}\left(k+q-1\right) \end{array}\right] \end{array}$$

Let $\bar{X}\left(k+q\right)=\left[\begin{array}{c} x^{\left(1\right)}\left(k+1\right)\\ x^{\left(1\right)}\left(k+2\right)\\ \vdots \\ x^{\left(1\right)}\left(k+q\right) \end{array}\right]$, $\bar{A}\left(k+q\right)=\left[\begin{array}{cccc} A(k) & A(k+1)A(k) & \cdots & \prod_{i=0}^{q-1}A(k+i) \end{array}\right]$, $\bar{W}\left(k+q\right)=\left[\begin{array}{c} w^{\left(1\right)}\left(k\right)\\ A\left(k+1\right)w^{\left(1\right)}\left(k\right)+w^{\left(1\right)}\left(k+1\right)\\ \vdots \\ \sum_{l=0}^{q-2}\prod_{i=0}^{q-2-l}A\left(k+i+1\right)w^{\left(1\right)}\left(k+l\right)+w^{\left(1\right)}\left(k+q-1\right) \end{array}\right]$. 

Accordingly, based on the established multi-step prediction state model, the multi-step measurement model is established. According to the obtained state model sequence, the measurement model sequence is obtained.

(8) $$\left\{\begin{array}{c} y\left(k+1\right)=H\left(k+1\right)x^{\left(1\right)}\left(k+1\right)+v\left(k+1\right)\\ y\left(k+2\right)=H\left(k+2\right)x^{\left(1\right)}\left(k+2\right)+v\left(k+2\right)\\ \vdots \\ y\left(k+q\right)=H\left(k+q\right)x^{\left(1\right)}\left(k+q\right)+v\left(k+q\right) \end{array}\right.$$

This can be expressed as

(9) $$\left[\begin{array}{c} y(k+1)\\ y(k+2)\\ \vdots \\ y(k+q) \end{array}\right]=\left[\begin{array}{cccc} H(k+1) & H(k+2) & \cdots & H(k+q) \end{array}\right]\left[\begin{array}{c} x^{\left(1\right)}\left(k+1\right)\\ x^{\left(1\right)}\left(k+2\right)\\ \vdots \\ x^{\left(1\right)}\left(k+q\right) \end{array}\right]+\left[\begin{array}{c} v(k)\\ v(k+1)\\ \vdots \\ v(k+q) \end{array}\right]$$

where H(·) is the measurement matrix.

The state at time *k* is sequentially combined into the measurement from time k+1 to time k+q, as shown in Equation (4).

(10) $$\begin{array}{c} y\left(k+1\right)=H\left(k+1\right)x^{\left(1\right)}\left(k+1\right)+v\left(k+1\right)\\ \begin{array}{c} y\left(k+2\right)=H\left(k+2\right)H\left(k+1\right)x^{\left(1\right)}\left(k+1\right)\\ +H(k+2)v(k+1)+v(k+2) \end{array}\\ \vdots \\ \begin{array}{c} y\left(k+q\right)=\prod_{i=0}^{q}H(k+i)x^{\left(1\right)}\left(k+1\right)\\ +\sum_{l=0}^{q-1}\prod_{i=0}^{q-1-l}H(k+i+1)v(k+l)+v\left(k+q\right) \end{array} \end{array}$$

Equation (4) is organized into a general matrix form, which is

(11) $$\begin{array}{cc} \left[\begin{array}{c} y(k+1)\\ y(k+2)\\ \vdots \\ y(k+q) \end{array}\right] & =\left[\begin{array}{cccc} H(k+1) & H(k+2)H(k+1) & \cdots & \prod_{i=0}^{q}H(k+i) \end{array}\right]x^{\left(1\right)}\left(k+1\right)\\ \; & +\left[\begin{array}{c} v(k+1)\\ H(k+1)v(k+1)+v(k+2)\\ \vdots \\ \sum_{l=0}^{q-1}\prod_{i=0}^{q-1-l}H(k+i)v(k+l)+v\left(k+q\right) \end{array}\right] \end{array}$$

Let $\bar{Y}\left(k+q\right)=\left[\begin{array}{c} y(k+1)\\ y(k+2)\\ \vdots \\ y(k+q) \end{array}\right]$, $\bar{H}\left(k+q\right)=\left[\begin{array}{cccc} H(k+1) & H(k+2)H(k+1) & \cdots & \prod_{i=0}^{q}H(k+i) \end{array}\right]$, $\bar{V}\left(k+q\right)=\left[\begin{array}{c} v(k+1)\\ H(k+1)v(k+1)+v(k+2)\\ \vdots \\ \sum_{l=0}^{q-1}\prod_{i=0}^{q-1-l}H(k+i)v(k+l)+v\left(k+q\right) \end{array}\right]$. 

The establishment of the above multi-step state model and measurement model immediately obtains the multi-step prediction model based on the one-step prediction mechanism model.

(12) $$\left\{\begin{array}{c} \bar{X}\left(k+q\right)=\bar{A}\left(k+q\right)x^{\left(1\right)}\left(k\right)+\bar{W}\left(k+q\right)\\ \bar{Y}\left(k+q\right)=\bar{H}\left(k+q\right)\bar{X}\left(k+q\right)+\bar{V}\left(k+q\right) \end{array}\right.$$

**Remark** **1.** *In the process of establishing the multi-step prediction model, the multi-step state transition matrix $\bar{A}\left(k+q\right)$ and multi-step measurement matrix $\bar{H}\left(k+q\right)$ need to be obtained by the iterative accumulation of A(·) and H(·). If the original state equation and measurement equation are linear, the linear dimension expansion processing is directly used. If it is nonlinear, this paper uses the first-order partial derivative method of EKF to process and finally forms a pseudo-linear filtering equation. $\bar{W}\left(k+q\right)$ and $\bar{V}\left(k+q\right)$ were derived from the above derivation, and their covariance $\bar{Q}\left(\cdot \right)$ and $\bar{R}\left(\cdot \right)$ are calculated according to the independent characteristics of Gaussian white noise.*

## 4. Multi-Step Prediction of Future Measurement

**Figure 1:**
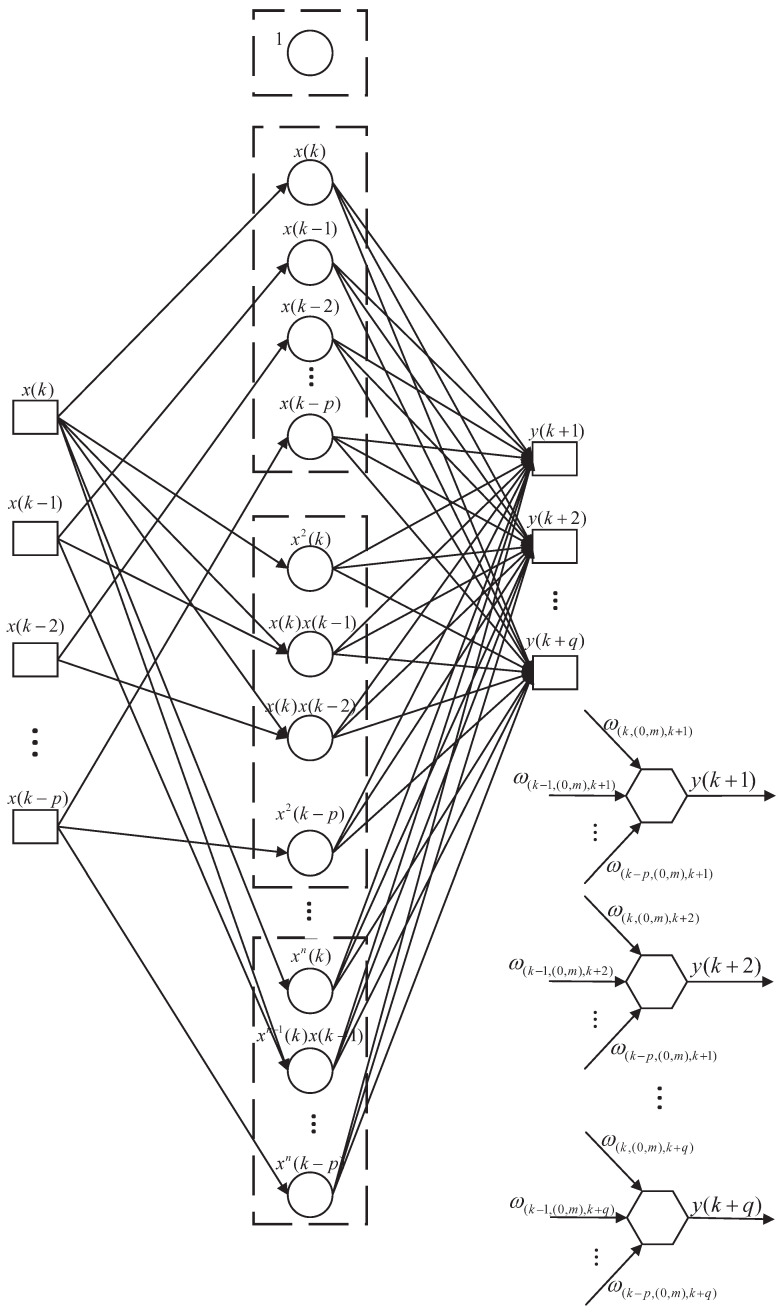
Multi-step prediction structure based on a multi-dimensional Taylor network.

Compared with the traditional neural network, on the one hand, the MTN structure expands the activation function of the hidden layer of the artificial neural network by Taylor series, and describes the general form of the output of the artificial neural network with the activation function after Taylor expansion, thus, serving as a theoretical basis for explaining the artificial neural network [33,34]. On the other hand, the polynomial function of MTN consists of some nonlinear and linear terms, and thus it can be used as a representation model of state dynamics. Due to the high degree of information correlation and coupling between adjacent moments, and in order to ensure that the amount of information to predict the future measurement is sufficient, we use the periodic multi-time past measurement to predict the future measurement. The network structure diagram of multi-step prediction of future measurements is shown in Figure 1.

The structure of the multi-step prediction multi-dimensional Taylor network model is a forward single middle layer, including an input layer, middle layer and output layer. Let the input layer have p+1 nodes, $X\left(k,k-p\right)={\left[x(k),x(k-1),\cdots ,x(k-p)\right]}^{T}$, Let the output layer have *q* nodes, $Y\left(k+q,k\right)={\left[y\left(k+1\right),y\left(k+2\right),\cdots ,y\left(k+q\right)\right]}^{T}$. The middle layer is the network processing layer, and the input data exist in the form of weighted summation of the power product units in the middle layer, where the weight variables of the output layer and the middle layer are represented by $\omega_{\left((k-p,k),(0,m),(k,k+q)\right)}$.

(13) $${\left[\begin{array}{cccc} \omega_{\left(k,(0,m),k+1\right)} & \omega_{\left(k-1,(0,m),k+1\right)} & \cdots & \omega_{\left(k-p,(0,m),k+1\right)}\\ \omega_{\left(k,(0,m),k+2\right)} & \omega_{\left(k-1,(0,m),k+2\right)} & \cdots & \omega_{\left(k-p,(0,m),k+2\right)}\\ \vdots & \vdots & \ddots & \vdots \\ \omega_{\left(k,(0,m),k+q\right)} & \omega_{\left(k-1,(0,m),k+q\right)} & \cdots & \omega_{\left(k-p,(0,m),k+q\right)} \end{array}\right]}_{(p+1)\times q}=\omega_{\left((k-p,k),(0,m),(k,k+q)\right)}$$

**Weierstrass** **Approximation** **Theorem:** *Continuous Functions on Closed Intervals Can Be Approximated Uniformly by Polynomial Series.*

**Lemma** **1.** *The continuous function $f(x_{1},x_{2},\cdots ,x_{n})$ defined on a closed interval can be approximated by $\sum_{t=1}^{N(n,m)}\omega_{t}\prod_{i=1}^{n}x_{i}^{\gamma_{t,i}}$, where N(n,m) is the total number of terms in the approximation expansion, and $\gamma_{t,i}$ is the power of the variable $x_{i}$ in the t−th product term in the expansion. From the Weierstrass approximation theorem and Lemma 1, the output results of MTN can be approximated by $\sum_{t=1}^{N(n,m)}\omega_{t}\prod_{i=1}^{n}x_{i}^{\gamma_{t,i}}$.*

For the multi-dimensional Taylor network with multiple nodes in the output layer, each output node can be written in the following matrix form:

(14) $$Y\left(k+q,k\right)={\left[y\left(k+1\right),y\left(k+2\right),\cdots ,y\left(k+q\right)\right]}^{T}=\omega_{\left((k-p,k),(0,m),(k,k+q)\right)}M_{node}\left(k-p,k\right)$$

where $M_{node}\left(k-p,k\right)$ is the node of MTN.

(15) $${\left[1,x\left(k\right),\cdots ,x\left(k-p\right),\cdots ,x^{n-1}\left(k\right)x\left(k-1\right),\cdots ,x^{n}\left(k-p\right)\right]}^{T}=M_{node}\left(k-p,k\right)$$

As there are similarities between the MTN structure and extreme learning, only the weight training from the middle layer to the output layer is needed. Therefore, the parameters in the MTN structure can also be solved by the least-square method, and thus there are:

(16) $$\min{∥\omega_{\left((k-p,k),(0,m),(k,k+q)\right)}M_{node}\left(k-p,k\right)∥}_{2}$$

The optimal solution is

(17) $$\omega_{\left((k-p,k),(0,m),(k,k+q)\right)}=Y\left(k+q,k\right)M_{node}{\left(k-p,k\right)}^{T}{\left[M_{node}\left(k-p,k\right)M_{node}{\left(k-p,k\right)}^{T}\right]}^{-1}$$

## 5. Filtering Estimation of Remaining Useful Life

**Figure 2:**
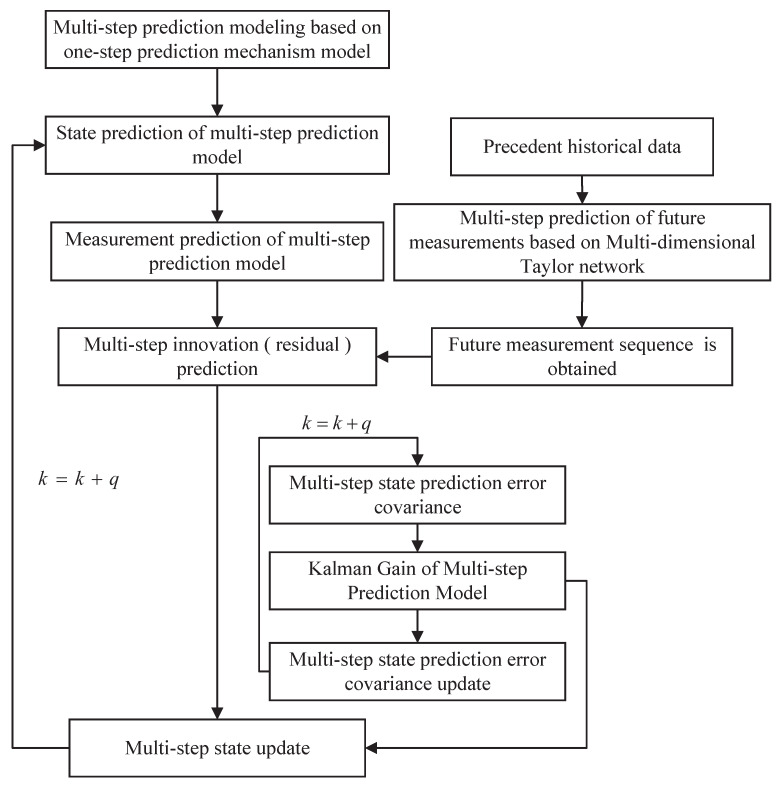
Flow chart of overall algorithm.

According to the establishment of the above multi-step expansion model and the realization of multi-step prediction of multi-dimensional Taylor network, their results are combined into the filtering estimation of remaining useful life. The flow chart is shown in Figure 2.

The time update process:

(1) The initial predictive value of the multi-step expansion model is obtained based on the initial state estimation x(k|k) and state transition matrix $\bar{A}\left(k+q|k\right)$ of the multi-step extended system.

(18) $$\bar{X}\left(k+q|k\right)=\bar{A}\left(k+q|k\right)x\left(k|k\right)$$

(2) According to the covariance matrix at time *k* of the multi-step expansion model and the variance of process noise, the prediction error covariance matrix is obtained.

(19) $$\bar{P}\left(k+q|k\right)=\bar{A}\left(k+q|k\right)\bar{P}\left(k|k\right){\bar{A}}^{T}\left(k+q|k\right)+\bar{Q}\left(k\right)$$

Measurement update process:

(3) According to the covariance matrix of the prediction error of the multi-step expansion model and the relevant information of the measured values, the gain matrix can be obtained.

(20) $$\bar{K}\left(k+q\right)=\bar{P}\left(k+q|k\right){\bar{H}}^{T}\left(k+q\right){\left[\bar{H}\left(k+q\right)\bar{P}\left(k+q|k\right){\bar{H}}^{T}\left(k+q\right)+\bar{R}\left(k+q\right)\right]}^{-1}$$

(4) According to the state prediction, the filtering gain $\bar{K}\left(k+q\right)$ and the error between the measured and predicted values of multi-step prediction, a new high-order extended Kalman filter is obtained.

(21) $$\bar{X}\left(k+q|k+q\right)=\bar{X}\left(k+q|k\right)+\bar{K}\left(k+q\right)\left[Y\left(k+q\right)-H\left(k+q\right)\bar{X}\left(k+q\right)\right]$$

(5) The error covariance matrix of the multi-step expansion model is calculated

(22) $$\bar{P}\left(k+q|k+q\right)=\left[I-\bar{K}\left(k+q\right)\bar{H}\left(k+q\right)\right]\bar{P}\left(k+q|k\right)$$

**Remark** **2.** *The estimation method of the RUL utilizes the idea of a high-order extended Kalman filter, which extends the high-order information to the new system for updating. Similarly, this paper incorporates multi-step information into the update of the new system. Most importantly, the sliding fixed step filtering update provides the possibility for a real-time update.*

## 6. Experimental Verification

### 6.1. Before Experience

The RUL model of the lithium-ion battery in this paper is set as follows:

(23) $$\left\{\begin{array}{c} a^{\left(1\right)}\left(k+1\right)=a^{\left(1\right)}\left(k\right)+w_{a}\left(k\right),w_{a}\left(k\right)∼N\left(0,s_{a}\right)\\ b^{\left(1\right)}\left(k+1\right)=b^{\left(1\right)}\left(k\right)+w_{b}\left(k\right),w_{b}\left(k\right)∼N\left(0,s_{b}\right)\\ c^{\left(1\right)}\left(k+1\right)=c^{\left(1\right)}\left(k\right)+w_{c}\left(k\right),w_{c}\left(k\right)∼N\left(0,s_{c}\right)\\ d^{\left(1\right)}\left(k+1\right)=d^{\left(1\right)}\left(k\right)+w_{d}\left(k\right),w_{d}\left(k\right)∼N\left(0,s_{d}\right)\\ Q\left(k\right)=a^{\left(1\right)}\left(k\right)e^{\left(b^{\left(1\right)}\left(k\right)\cdot k\right)}+c^{\left(1\right)}\left(k\right)e^{\left(d^{\left(1\right)}\left(k\right)\cdot k\right)}+v\left(k\right) \end{array}\right.$$

where $x^{\left(1\right)}\left(k\right)={\left[a^{\left(1\right)}\left(k\right),b^{\left(1\right)}\left(k\right),c^{\left(1\right)}\left(k\right),d^{\left(1\right)}\left(k\right)\right]}^{T}$ is the parameter in the model, and there is Gaussian white noise with mean value of zero and a variance of in each parameter, $s^{\left(1\right)}=\left[s_{a},s_{b},s_{c},s_{d}\right]$; *Q* is the battery capacity; *k* is the charge and discharge cycle; and *v* is Gaussian white noise with mean value of zero and a variance of $s_{v}$—that is $v\left(k\right)∼N\left(0,s_{v}\right)$.

**Remark** **3.** *This model is an empirical model of a lithium-ion battery and is fitted based on the degradation characteristics with no special physical characteristics. In this experiment, in order to compare the effectiveness of different methods in a fair way, the prediction methods based on this model are the same model parameters, which contain very small Gaussian white noise, and its error can be ignored.*

**Figure 3:**
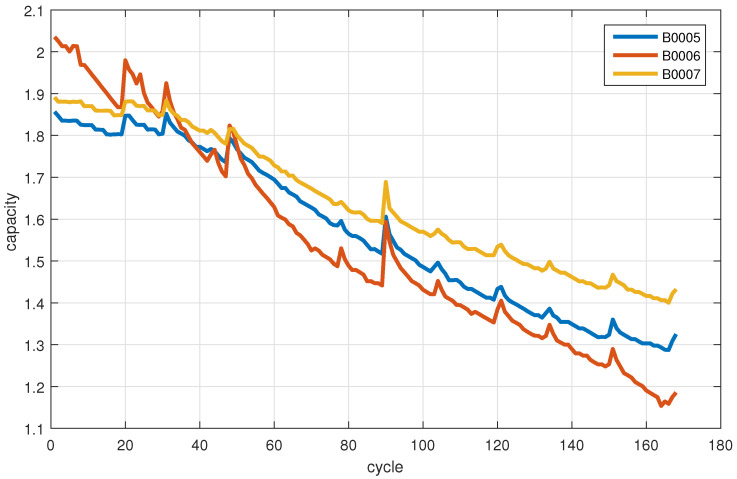
The relationship between the capacity and charge–discharge cycle of four lithium-ion batteries in the NASA PCoE laboratory.

In this paper, the lithium-ion battery data set provided by NASA PCoE laboratory is used for simulation verification. We extract the data relationship between the battery capacity and charge–discharge cycle of B0005, B0006 and B0007 batteries as shown in Figure 3. In addition, the initial values of the model parameters are provided by Table 1.

It can be seen from Figure 3 that, with the increase of charging and discharging cycles, the RUL of lithium-ion battery declines. In order to verify that the prediction effect of the proposed multi-step prediction method is not weaker than the existing one-step prediction method. We conducted comparative tests on different types of lithium-ion batteries. Since this method and the existing one-step prediction method combine network training based on historical data, we started the post-prediction from the 50th charge–discharge cycle and the 100th charge–discharge cycle, respectively. At the same time, the known data before *k* was used to predict the RUL after *k*.

### 6.2. Introduction of Various Methods

Since the comparative experiments in this paper involve three types of methods, in order to ensure the fairness and observability of the experimental data, the experimental part based on the model method uses the unscented Kalman filter ( UKF ). The experimental part based on data-driven method uses correlation vector machine ( RVM ), convolutional neural network ( CNN ) and long short-term memory network ( LSTM ) in a deep-learning method. The hybrid method combines UKF with RVM algorithm and the hybrid method proposed in this paper. The method proposed in this paper is a hybrid method combining a high-order extended Kalman filter and multi-step prediction by MTN. The following explains the application of the above method in this experiment.

UKF: According to the basic model given by Equation (23), the unscented Kalman filter is used to predict the RUL of lithium-ion batteries

RVM: This is the use of known lithium-ion battery data to train the RVM model to predict its RUL.

CNN: This is the use of known lithium-ion battery data to train the CNN model to predict its RUL.

LSTM: This is the use of known lithium-ion battery data to train the LSTM model to predict its RUL.

UKF+RVM: The RVM algorithm is introduced into the innovation update part of unscented Kalman filter to predict the measurements, and then the RUL predicted by the filter is obtained [28].

MEKF+MTNMP (q = 1): The innovation update part of one-step composite high-order extended Kalman filter introduces the measured predictive value of one-step prediction of the MTN.

MEKF+MTNMP (q = 2): The innovation update part of the two-step composite high-order extended Kalman filter introduces the measurement and prediction value of two-step prediction of the MTN.

MEKF+MTNMP (q = 3): The innovation update part of the three-step composite high-order extended Kalman filter introduces the measured predictive value of the three-step prediction of the MTN.

### 6.3. Comparative Experiments

**Figure 4:**
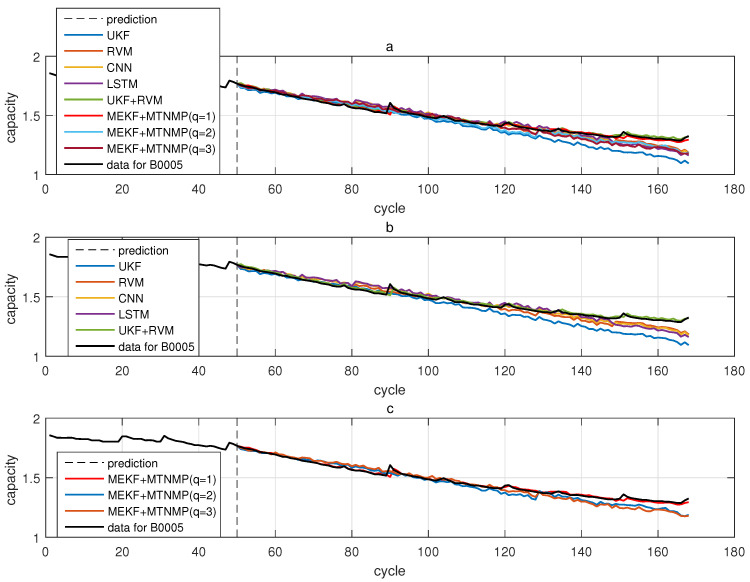
The simulation diagram of B0005 predicted from the 50th time. (**a**) is the experimental diagram of all methods, (**b**) is other methods used for comparison in (**a**,**c**) is the method proposed in this paper in (**a**).

**Figure 5:**
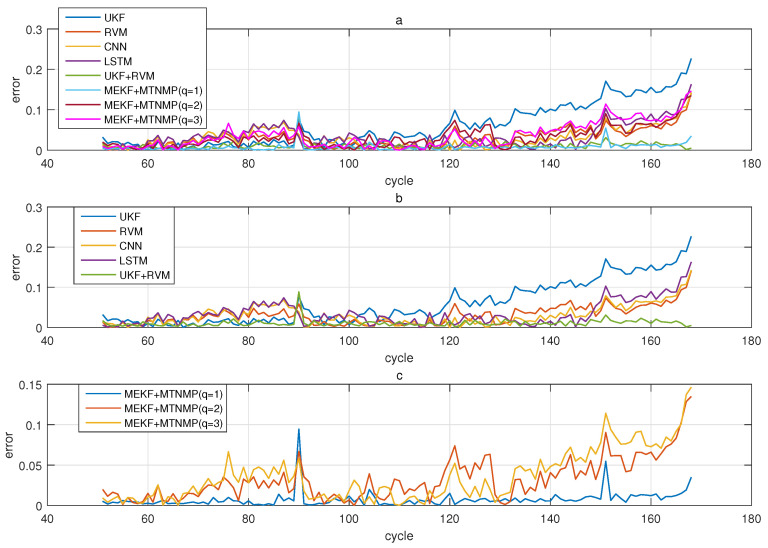
The simulation error diagram of B0005 predicted from the 50th time. (**a**) is the experimental error of all methods, (**b**) is the error of other methods used for comparison in (**a**), and (**c**) is the error of the method proposed in this paper in (**a**).

**Figure 6:**
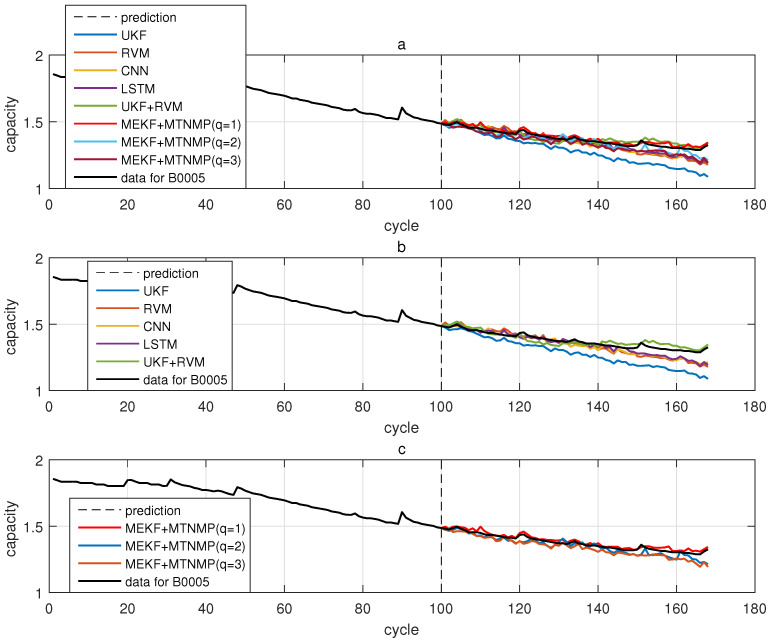
The simulation diagram of B0005 predicted from the 100th time. (**a**) is the experimental diagram of all methods, (**b**) is other methods used for comparison in (**a**,**c**) is the method proposed in this paper in (**a**).

**Figure 7:**
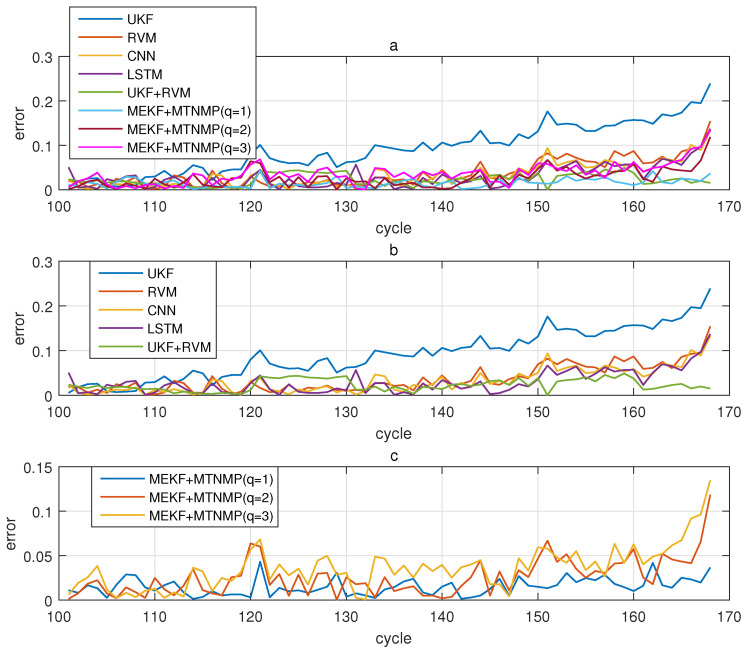
The simulation error diagram of B0005 predicted from the 100th time. (**a**) is the experimental error of all methods, (**b**) is the error of other methods used for comparison in (**a**), and (**c**) is the error of the method proposed in this paper in (**a**).

**Figure 8:**
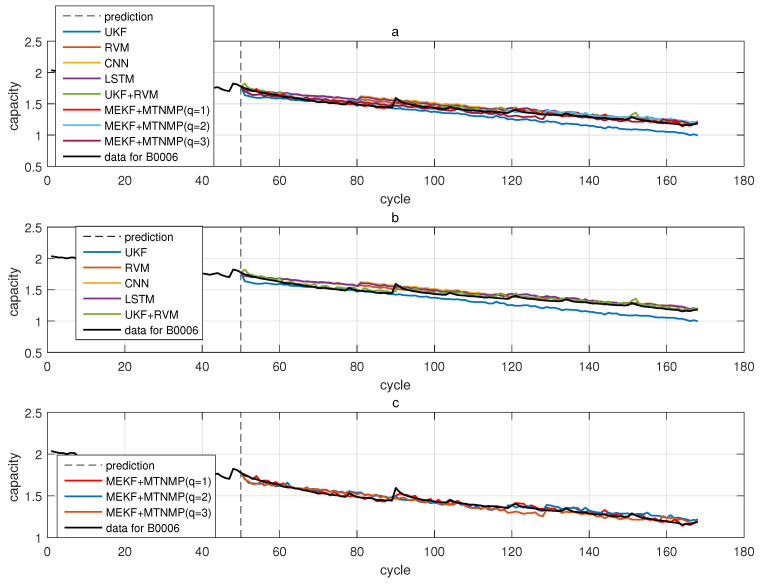
The simulation diagram of B0006 predicted from the 50th time. (**a**) is the experimental diagram of all methods, (**b**) is other methods used for comparison in (**a**,**c**) is the method proposed in this paper in (**a**).

**Figure 9:**
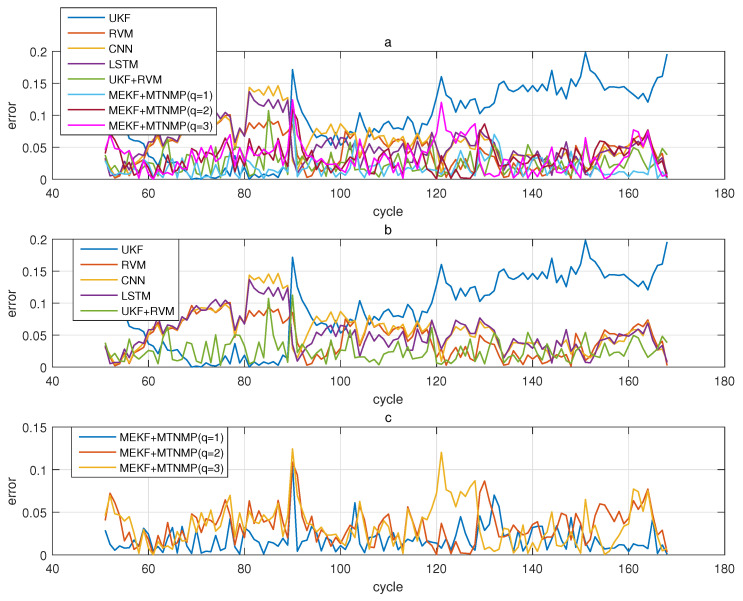
The simulation error diagram of B0006 predicted from the 50th time. (**a**) is the experimental error of all methods, (**b**) is the error of other methods used for comparison in (**a**), and (**c**) is the error of the method proposed in this paper in (**a**).

**Figure 10:**
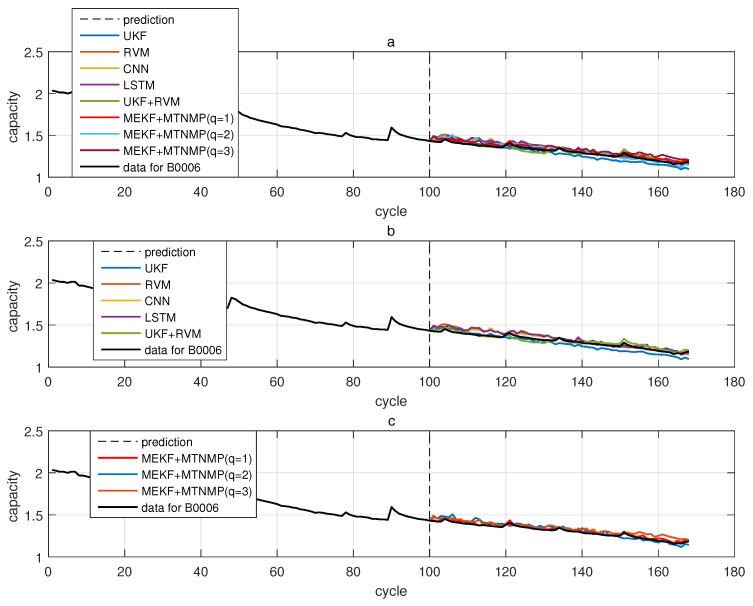
The simulation diagram of B0006 predicted from the 100th time. (**a**) is the experimental diagram of all methods, (**b**) is other methods used for comparison in (**a**,**c**) is the method proposed in this paper in (**a**).

**Figure 11:**
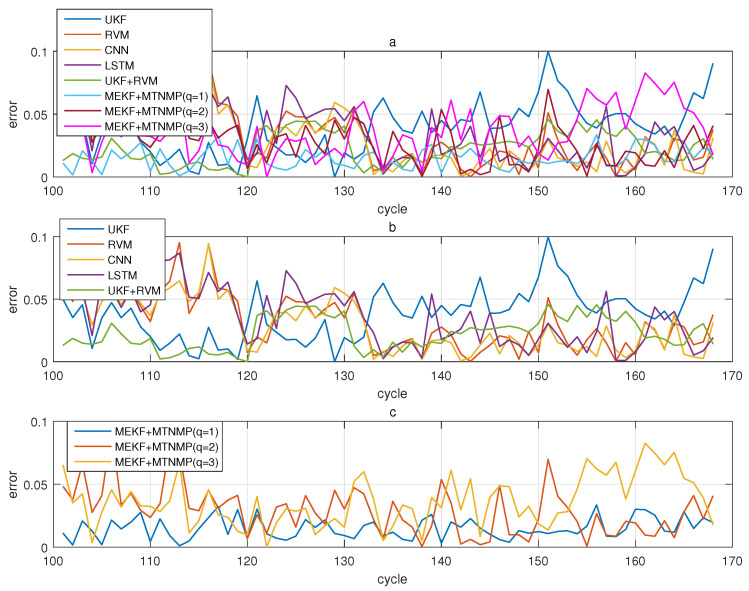
The simulation error diagram of B0006 predicted from the 100th time. (**a**) is the experimental error of all methods, (**b**) is the error of other methods used for comparison in (**a**), and (**c**) is the error of the method proposed in this paper in (**a**).

**Figure 12:**
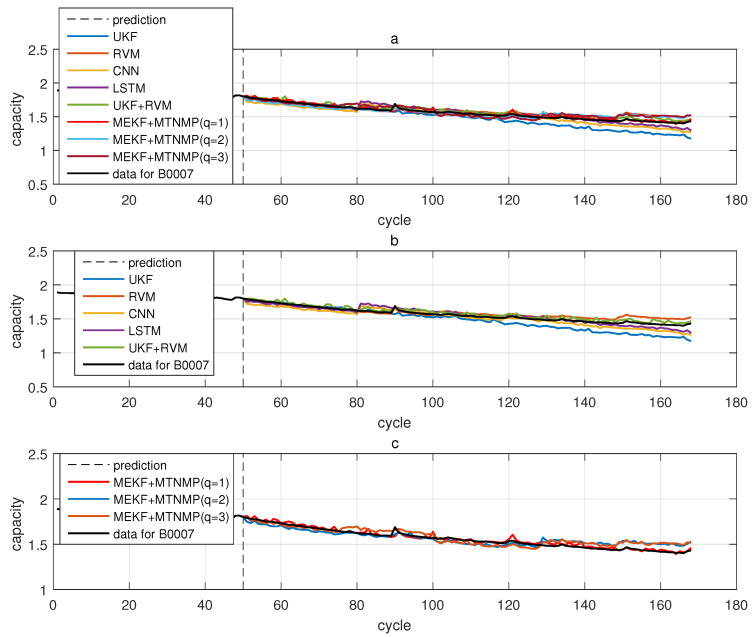
The simulation diagram of B0007 predicted from the 50th time. (**a**) is the experimental diagram of all methods, (**b**) is other methods used for comparison in (**a**,**c**) is the method proposed in this paper in (**a**).

**Figure 13:**
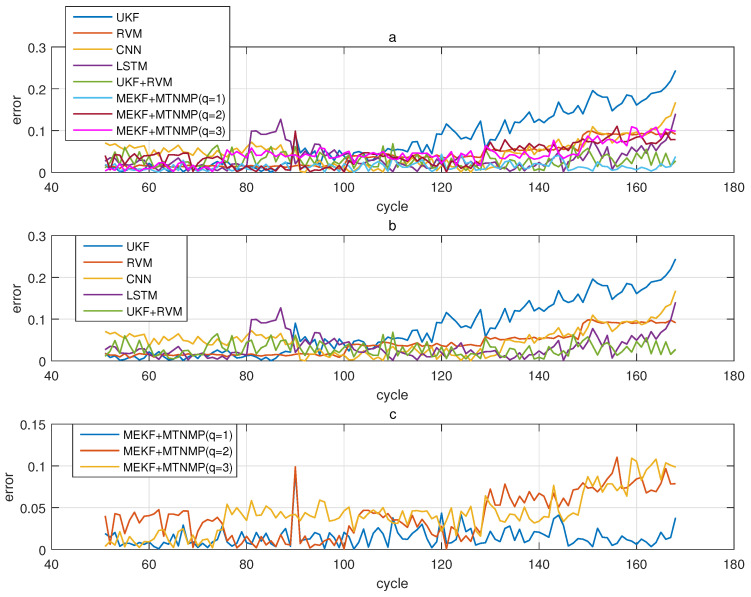
The simulation error diagram of B0007 predicted from the 50th time. (**a**) is the experimental error of all methods, (**b**) is the error of other methods used for comparison in (**a**), and (**c**) is the error of the method proposed in this paper in (**a**).

**Figure 14:**
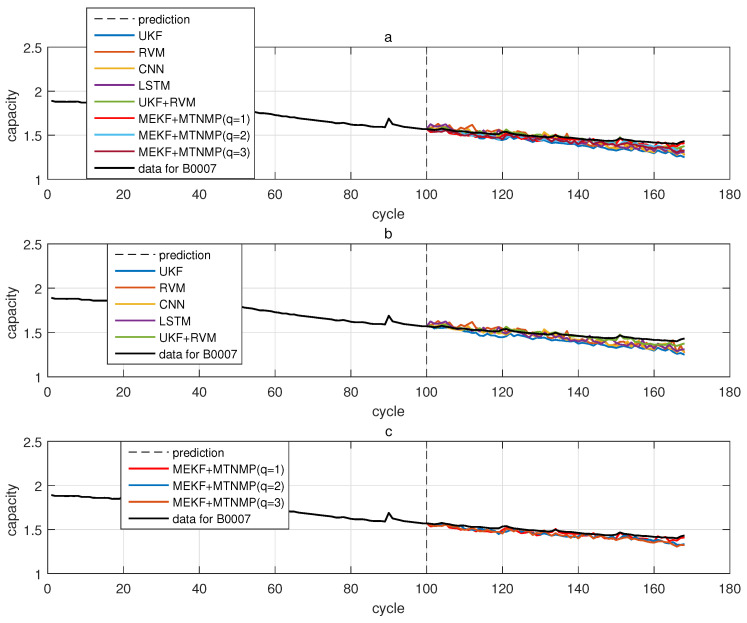
The simulation diagram of B0007 predicted from the 100th time. (**a**) is the experimental diagram of all methods, (**b**) is other methods used for comparison in (**a**,**c**) is the method proposed in this paper in (**a**).

**Figure 15:**
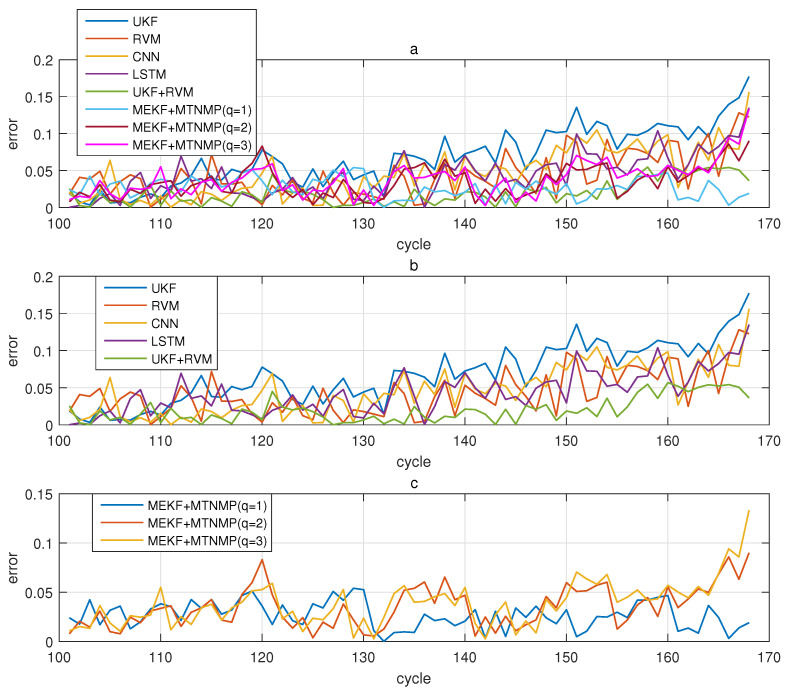
The simulation error diagram of B0007 predicted from the 100th time. (**a**) is the experimental error of all methods, (**b**) is the error of other methods used for comparison in (**a**), and (**c**) is the error of the method proposed in this paper in (**a**).

The comparative experiments in this section record the performance of the above eight methods in three lithium-ion battery data sets. Figure 4, Figure 5, Figure 6 and Figure 7 are the performance of eight methods in the B0005 lithium-ion battery data set. Figure 5 and Figure 7 are the absolute error between the predicted RUL and the real RUL. Table 2 records their mean square error, and Table 3 shows the performance comparison between the methods in the form of percentage. Figure 8, Figure 9, Figure 10 and Figure 11 are the performance of the eight methods in the B0006 lithium-ion battery data set. Table 4 records their mean square error, and Table 5 shows the performance comparison between the methods in the form of percentage. Figure 12, Figure 13, Figure 14 and Figure 15 are the performance of eight methods in the B0007 lithium-ion battery data set. Table 6 records their mean square error, and Table 7 shows the performance comparison between the methods in the form of percentage. Finally, Table 8 shows the effect changes from the beginning of prediction at k=100 to the beginning of prediction at k=50—that is, to compare the impact on each method when the amount of data increases slightly.

Through the mean square error recorded in Table 2, Table 4 and Table 6, it is not difficult to see the effectiveness of the proposed method and the effect of this method is generally better than that of UKF. The UKF+RVM algorithm shows the strong performance of the existing one-step prediction hybrid method; however, the proposed method is not inferior to UKF+RVM in one-step prediction.

At the same time, the percentage data in Table 3, Table 5 and Table 7 intuitively show that this method has more than 50% performance than other methods. As for the two-step prediction, the three-step prediction does not lose accuracy while achieving multi-step prediction. Although the accuracy here is less than that of UKF+RVM in the one-step prediction method, it is also higher than that of RVM, CNN and LSTM. The main contribution of this method is proven from the mean square error and comparison:

- The proposed method can effectively predict the RUL of lithium-ion batteries.
- Compared with the model-based filtering method, this method can effectively improve the accuracy from 66.13% to 97.67%.
- The one-step prediction accuracy is 12.95% to 63.42% higher than UKF+RVM, which was proposed in the literature [28].

The robustness of the proposed method is further illustrated in Table 8. The three methods based on data-driven methods do not have a stable prediction effect on the data with a lower amplitude increase. While the proposed method increases the prediction step, the training data with less amplitude increases the prediction effect. The two-step prediction and three-step prediction of this method increased by 35.29%, 25.00% and 20.83% as well as 31.58%, 5.88% and 16.00% in the three data sets, respectively. On the other hand, this method has good anti-interference ability and robustness to the change of data set. We also show that, compared with the data-driven method, relying on a large number of data and training models, this method was more prominent in real-time updates and small sample requirements. These prove the main contributions of this method:

- The multi-step prediction method improved the prediction accuracy by 5.88% to 35.29% when the data increased slightly.
- This multi-step prediction had good robustness.

From these experiments, we found that the model-only UKF had a poor effect in the one-step prediction of three lithium-ion batteries because the fitting model based on double exponential energy attenuation approximated the degradation effect of lithium-ion battery due to the use of cycle; therefore, the filtering results included the fitting error and the prediction error of the unscented Kalman filter. Based on the data of RVM, CNN and LSTM, there was no obvious trend in the prediction effect at k=100 and k=50, which reflects that the data-driven prediction method relied heavily on the quantity and quality of historical data and was not suitable for small sample size and real-time update.

Model-based and data-driven methods showed good prediction accuracy but could only complete one-step prediction. The multi-step prediction method proposed in this paper with one-step and two-step prediction accuracy were trustworthy in the process of one-step to three-step prediction. Although three-step prediction accuracy was not as good as one-step and two-step prediction, as long as the historical data is sufficient, its prediction accuracy was still acceptable.

## 7. Conclusions and Future Work

In this paper, we proposed a real-time updating high-order extended Kalman filter method for lithium-ion batteries based on fixed-step life prediction.

- The most important contribution of this method is to realize the use of past values to predict multiple future time values—that is, multi-step measurement based on one-step prediction.
- Secondly, dynamic modeling of a multi-step prediction model that meets the requirements of the Kalman filter was realized, and the corresponding step filter was established.
- Thirdly, the establishment of fixed-step prediction based on multi-dimensional Taylor network provides multiple future output values for the filtering process of multi-step prediction to, thus, realize the real-time updating of prediction innovation.

On the basis of multi-step prediction, the prediction accuracy of this method was not reduced, and it retained excellent robustness under the support of less historical data. Although this paper contributes to the field of multi-step prediction of the RUL of lithium-ion batteries, there remains future work to be performed. The modeling error of multi-step prediction increases with the increase of the prediction step size, which affects the prediction accuracy. This problem can be seen from the experimental results. The mean square error of the prediction results increases with the increase of prediction step. The multi-step prediction in this paper was conducted in the ideal environment of Gaussian white noise, and the multi-step prediction dynamic modeling in a non-Gaussian environment is also a problem to be solved.

## Figures and Tables

**Table 1:** The initial values of the model parameters.

Battery ID	$a^{\left(1\right)}$	$b^{\left(1\right)}$	$c^{\left(1\right)}$	$d^{\left(1\right)}$
B0005	2.4081	−0.0045	−0.5899	−0.0231
B0006	2.4168	−0.0076	1.2677	−0.0016
B0007	2.3574	−0.0018	0.2664	−0.0017

**Table 2:** Experimental results of B0005 lithium-ion battery.

Methods	MSE When k=50	MSE When k=100
UKF	0.0066	0.0114
RVM	0.0014	0.0022
CNN	0.0011	0.0018
LSTM	0.0016	0.0014
UKF+RVM	2.2481 × 10${}^{-4}$	7.0365 × 10${}^{-4}$
MEKF+MTNMP (q = 1)	1.5365 × 10${}^{-4}$	3.3435 × 10${}^{-4}$
MEKF+MTNMP (q = 2)	0.0017	0.0011
MEKF+MTNMP (q = 3)	0.0019	0.0013

**Table 3:** Comparison of methods in the B0005 experiment.

Method to Be Compared	MEKF+MTNMP (q = 1)		MEKF+MTNMP (q = 2)		MEKF+MTNMP (q = 3)	
	When k=50	When k=100	When k=50	When k=100	When k=50	When k=100
UKF	97.67%	97.07%	74.24%	90.35%	71.21%	86.60%
RVM	89.03%	84.80%	−21.43%	50.00%	−35.71%	40.91%
CNN	86.03%	81.43%	−54.55%	38.89%	−72.73%	27.78%
LSTM	90.40%	76.12%	−6.25%	21.43%	−18.75%	7.14%
UKF+RVM	31.65%	52.48%	−656.19%	−56.33%	−745.16%	97.67%
MEKF+MTNMP (q = 1)	-	-	−1006.41%	−229.00%	−1136.58%	−288.81%
MEKF+MTNMP (q = 2)	90.96%	69.60%	-	-	−11.76%	−18.18%
MEKF+MTNMP (q = 3)	91.91%	74.28%	10.53%	15.38%	-	-

**Table 4:** Experimental results of the B0006 lithium-ion battery.

Methods	MSE When k=50	MSE When k=100
UKF	0.0110	0.0069
RVM	0.0027	0.0012
CNN	0.0033	0.0012
LSTM	0.0026	0.0018
UKF+RVM	0.0012	7.2634 × 10${}^{-4}$
MEKF+MTNMP (q = 1)	4.3902 × 10${}^{-4}$	3.2411 × 10${}^{-4}$
MEKF+MTNMP (q = 2)	0.0016	0.0012
MEKF+MTNMP (q = 3)	0.0017	0.0016

**Table 5:** Comparison of methods in the B0006 experiment.

Method to Be Compared	MEKF+MTNMP (q = 1)		MEKF+MTNMP (q = 2)		MEKF+MTNMP (q = 3)	
	When k=50	When k=100	When k=50	When k=100	When k=50	When k=100
UKF	96.01%	95.30%	85.45%	82.61%	84.55%	76.81%
RVM	83.74%	72.99%	40.74%	0.00%	37.04%	−33.33%
CNN	86.70%	72.99%	51.52%	0.00%	48.48%	−33.33%
LSTM	83.11%	81.99%	38.46%	33.33%	34.62%	11.11%
UKF+RVM	63.42%	55.38%	−33.33%	−65.21%	−41.67%	−120.28%
MEKF+MTNMP (q = 1)	-	-	−264.45%	−270.24%	−287.23%	−393.66%
MEKF+MTNMP (q = 2)	72.56%	72.99%	-	-	−6.25%	−33.33%
MEKF+MTNMP (q = 3)	74.18%	79.74%	5.88%	25.00%	-	-

**Table 6:** Experimental results of the B0007 lithium-ion battery.

Methods	MSE When k=50	MSE When k=100
UKF	0.0100	0.0062
RVM	0.0024	0.0030
CNN	0.0034	0.0033
LSTM	0.0034	0.0035
UKF+RVM	0.0011	7.8800 × 10${}^{-4}$
MEKF+MTNMP (q = 1)	4.6750 × 10${}^{-4}$	6.8594 × 10${}^{-4}$
MEKF+MTNMP (q = 2)	0.0024	0.0019
MEKF+MTNMP (q = 3)	0.0025	0.0021

**Table 7:** Comparison of methods in the B0007 experiment.

Method to Be Compared	MEKF+MTNMP (q = 1)		MEKF+MTNMP (q = 2)		MEKF+MTNMP (q = 3)	
	When k=50	When k=100	When k=50	When k=100	When k=50	When k=100
UKF	95.33%	88.94%	76.00%	69.35%	75.00%	66.13%
RVM	80.52%	77.14%	0.00%	36.67%	−4.17%	30.00%
CNN	86.25%	79.21%	29.41%	42.42%	26.47%	36.36%
LSTM	86.25%	80.40%	29.41%	45.71%	26.47%	40.00%
UKF+RVM	57.50%	12.95%	−118.18%	−141.12%	−127.27%	−166.50%
MEKF+MTNMP (q = 1)	-	-	−413.37%	−176.99%	−434.76%	−206.15%
MEKF+MTNMP (q = 2)	80.52%	63.90%	-	-	−4.17%	−10.53%
MEKF+MTNMP (q = 3)	81.30%	67.34%	4.00%	9.52%	-	-

**Table 8:** The optimization degree of k=100 relative to k=50.

Methods	Improved in the B0005	Improved in the B0006	Improved in the B0007
UKF	-	-	-
RVM	−57.14%	55.56%	−25.00%
CNN	−63.64%	63.64%	2.94%
LSTM	12.50%	30.77%	−2.94%
UKF+RVM	−213.00%	39.47%	28.36%
MEKF+MTNMP (q = 1)	−117.60%	26.17%	−46.73%
MEKF+MTNMP (q = 2)	35.29%	25.00%	20.83%
MEKF+MTNMP (q = 3)	31.58%	5.88%	16.00%

## Data Availability

Not applicable.
